# Effects of tirzepatide therapy on body weight and body composition in adults with overweight and obesity

**DOI:** 10.3389/fendo.2026.1834580

**Published:** 2026-07-08

**Authors:** Haley Corso, Austin J. Graybeal, Emily Hoelscher, Sydney Roberson, Briana Curran, Catherine Johnson, Kelly E. Johnson

**Affiliations:** 1 Department of Kinesiology, Coastal Carolina University, Conway, SC, United States; 2 Department of Kinesiology, Harris College of Nursing and Health Sciences, Texas Christian University, Fort Worth, TX, United States; 3 Precision Medical Care LLC, Clarendon Hills, IL, United States

**Keywords:** body composition, fat mass, fat−free mass, GLP-1, obesity, weight loss

## Abstract

Obesity is a growing public health concern associated with increased risk of chronic diseases, including type 2 diabetes and cardiovascular disease. Glucagon-like peptide-1 receptor agonists (GLP-1RAs) have become a key therapeutic option for obesity management, yet real-world evidence describing short-term changes in body composition, particularly with newer agents such as tirzepatide, remains limited. This retrospective study examined changes in body weight, body mass index (BMI), and body composition among adults with obesity receiving GLP-1 receptor agonist therapy within a clinical obesity medicine program that included individualized lifestyle guidance. Thirty-five adults (53.4 ± 7.8 years; BMI 37.2 ± 9.16 kg/m²) receiving tirzepatide were evaluated. Therapy was initiated at 2.5 mg or 5 mg once weekly, with scaffolded dose escalation up to 15 mg based on tolerance and clinical response. Body weight was measured using standard procedures, and body composition was assessed with bioelectrical impedance analysis (InBody770) at baseline and follow-up visits. Linear mixed models and ANOVA were used to examine effects of time, sex, and baseline weight status (p < 0.050).GLP-1RA therapy resulted in significant reductions in body weight (−31.1 kg; −27.8%), BMI (−10.4 kg/m²; −27.8%), body fat percentage (−16.2%; −37.6%), fat mass (−26.4 kg; −54.2%), fat-free mass (−4.5 kg; −7.3%), skeletal muscle mass (−2.8 kg; −8.3%), and total body water (−3.5 kg; −7.7%) (all p < 0.001). Weight loss was predominantly attributable to fat mass, comprising 85.7% (95% CI: 81.9, 89.5) of total weight loss, with smaller contributions from fat-free and skeletal muscle mass. Reductions in extracellular (−2.6 kg; −9.5%) and intracellular water (−1.3 kg; −7.4%) were observed; however, fluid distribution indices did not change significantly over time.Sex-specific analyses showed females experienced greater percentage reductions in fat mass, fat-free mass, skeletal muscle mass, and total body water compared to males, while males exhibited greater reductions in fluid ratios (p < 0.05). Participants with higher baseline BMI demonstrated significantly greater percentage reductions in weight, BMI, fat mass, and body fat percentage compared to those with lower baseline BMI (p < 0.05), although post-treatment values were not significantly different between groups.

## Introduction

Obesity remains a major global public health challenge, with prevalence increasing steadily over recent decades and contributing to substantial medical, social, and economic burden (1, 2). Excess body weight is strongly associated with chronic conditions including type 2 diabetes mellitus, cardiovascular disease, certain cancers, and premature mortality (3–5).

Although dietary modification, behavioral strategies, and increased physical activity are foundational components of obesity management, long-term weight maintenance remains difficult for many individuals (6, 7). Physiological adaptations to weight loss, including changes in energy expenditure, appetite-regulating hormones, and metabolic efficiency, often contribute to weight regain. Consequently, there has been increasing interest in pharmacologic therapies that complement lifestyle interventions to support sustained weight reduction and improve obesity-related comorbidities.

Glucagon-like peptide-1 receptor agonists (GLP-1RAs) have emerged as an important advancement in obesity treatment. Initially developed for glycemic control in type 2 diabetes, these agents promote satiety, delay gastric emptying, and enhance glucose-dependent insulin secretion (8). Semaglutide, a once-weekly GLP-1 receptor agonist, has demonstrated mean weight reductions of approximately 15–16% in adults with overweight or obesity in large clinical trials (9). Tirzepatide, a dual glucose-dependent insulinotropic polypeptide/glucagon-like peptide-1 (GIP/GLP-1) receptor agonist, represents a newer therapeutic approach that combines the metabolic effects of both incretin pathways. This dual mechanism enhances insulin secretion, improves insulin sensitivity, delays gastric emptying, and suppresses appetite (10, 11). In addition to robust weight reduction, tirzepatide has demonstrated improvements in glycated hemoglobin, blood pressure, and lipid profiles, indicating broader cardiometabolic benefits (5, 12). In the SURMOUNT-1 trial, participants without diabetes achieved weight reductions of up to 22.5%, representing a significant advance compared to earlier anti-obesity medications (5).

However, changes in body weight alone do not fully characterize the physiological effects of pharmacologically induced weight loss. Body composition—including fat mass (kg), fat-free mass (kg), skeletal muscle mass (kg), and total body water (kg) and its compartments—is a critical determinant of metabolic health. Excessive reductions in lean tissue may negatively affect resting energy expenditure, physical function, and long-term weight stability and have been linked to sarcopenic obesity and adverse clinical outcomes (13, 14). Advanced imaging and body composition analyses have begun to quantify these changes in clinical trials. In a substudy of SURMOUNT-1, tirzepatide produced a 21.3% reduction in body weight over 72 weeks, with approximately three-quarters of weight loss attributable to fat mass and one-quarter to lean mass, indicating that losses in lean tissue remain clinically relevant and warrant monitoring (15).

Despite the demonstrated efficacy of GLP-1RA-based therapies, real-world data on longitudinal changes in body composition remain limited, particularly for dual GIP/GLP-1 receptor agonists such as tirzepatide. This gap is clinically important because total weight loss does not distinguish between beneficial reductions in fat mass and potentially detrimental losses of lean tissue or alterations in body water compartments. Bioelectrical impedance analysis (BIA) provides a practical and accessible outpatient method for estimating multiple body composition variables, including fat mass (kg), fat-free mass (kg), total body water (kg), extracellular water (kg), and intracellular water (kg). Prior studies using BIA suggest that weight loss with GLP-1-based therapies is largely driven by reductions in fat mass, with comparatively smaller decreases in lean tissue, while also demonstrating improvements in metabolic parameters (16, 17). These measurements may offer additional clinical value by identifying patients at risk for disproportionate lean mass loss and informing individualized management strategies.

The purpose of this retrospective study was to examine real-world longitudinal follow-up in routine care changes in body weight, body mass index, body fat percentage, fat mass, fat free mass, and skeletal muscle mass among adults with obesity receiving a dual GLP-1/GIP agonist, including tirzepatide, in a real-world clinical setting.

We hypothesized that treatment under specialist guided care with consistent dietary and exercise counseling would produce clinically meaningful weight loss characterized primarily by reductions in fat mass with relative preservation of fat free mass and skeletal muscle mass over the early course of therapy. We further hypothesized that bioelectrical impedance analysis would identify patients at risk for disproportionate lean mass loss and thereby inform timely adjustments to nutrition and resistance training strategies.

## Materials and methods

The study employed a retrospective, observational design to evaluate changes in body composition among adults undergoing dual agonist pharmacologic obesity treatment. Clinical data were extracted from electronic health records maintained at Precision Medical Care LLC for the period between March 2019 and December 31, 2024. All procedures followed the ethical standards of the Declaration of Helsinki, adhered to Good Pharmacoepidemiology Practices, and complied with relevant U.S. regulatory requirements. The Institutional Review Board of Coastal Carolina University approved the study protocol (IRB #2025.122−C−1; approval date January 14, 2026). Prior to analysis, all data were de−identified according to the HIPAA Privacy Rule’s Expert Determination method. All patients were managed by a Certified Obesity Medicine Specialist and received diet and exercise counseling at every visit. Bioelectrical impedance analysis was incorporated into routine monitoring alongside standard anthropometrics to provide a comprehensive evaluation of physiologic changes during pharmacologic weight loss.

A total of 35 adults between the ages of 41-75 (53.4 ± 7.8 years) with overweight and obesity (BMI 26.9-60.8 kg/m²) were included in the analysis. All participants were receiving routine obesity management at Precision Medical Care LLC and met inclusion criteria based on the availability of complete baseline and follow−up body composition data. Because the study utilized pre−existing, de−identified records, informed consent requirements followed institutional guidelines for secondary data use. Although the analysis was retrospective, all clinical measurements had been collected under standardized procedures. Prior to baseline body composition testing, patients were provided written instructions 48 hours in advance outlining required pre−assessment conditions. To ensure accuracy of bioelectrical impedance analysis (BIA), they were instructed to avoid alcohol consumption for 48 hours, maintain normal hydration, and abstain from exercise, meals, or caloric beverages for 12 hours prior to testing, consistent with manufacturer recommendations.

Body weight and composition were measured during routine clinical visits, beginning at treatment initiation at each visit. Body weight was assessed using a calibrated clinical scale, and height was recorded at baseline to calculate BMI. Body composition was evaluated using a multi−frequency BIA device (InBody770, InBody Japan), which provided estimates of BF%, FM, FFM, SMM, the SMM−to−age index, dry lean mass (DLM), TBW, ECW, and intracellular water (ICW). All measurements were obtained following the manufacturer’s standardized protocols. Although SMM estimates overlap with FFM, it is separate and defined as the functional muscle tissue attached to bone. SMM estimates using this BIA device is calculated using a proprietary equation unknown to the researchers and therefore, is not presented here. SMM-to-age index is calculated as SMM divided by age and is used to characterize sarcopenia. DLM is calculated as FFM minus TBW and refers to the body’s protein and mineral content.

Following baseline assessment, patients-initiated treatment with the GLP−1 receptor agonist tirzepatide as part of routine clinical care. Therapy was initiated at either 2.5 mg or 5 mg administered once weekly, with subsequent dose modification and escalation up to a maximum of 15 mg based on medication tolerance and individual clinical response.

Clinical response was assessed during regular follow−up visits with an obesity medicine physician and was determined through a combination of medication management review, annual biomarker assessment, and repeated body composition measurements each visits depending on appointment recommendation set by patient and physician. These visits were used to guide clinical decision−making regarding treatment modification and dose escalation; however, the primary study outcomes were evaluated at pre−treatment baseline and post−treatment follow−up rather than as longitudinal monthly measures.

Post treatment measurements were obtained at a consistent follow up time point of approximately 81 weeks, corresponding to the final scheduled clinical assessment for all participants. Therefore, the study reflects a fixed pre post design with uniform follow up duration across the cohort rather than variable or last observation time points. This approach minimizes heterogeneity in treatment exposure duration and ensures comparability of baseline and post treatment outcomes.

Complete data were available for all participants at both baseline and follow up, and no missing data were present for the variables included in the analysis. As a result, no imputation procedures were required. All participants remained on tirzepatide therapy through the follow up assessment based on medical record verification. Formal quantitative measures of medication adherence were not available; however, continued prescription and active participation in clinical follow up were confirmed for all individuals at the time of outcome measurement.

### Statistical analyses

Using a moderate effect size (f: 0.25) and correlation between measures (r: 0.50) for a within-between (two timepoints; two groups) interaction repeated measures ANOVA, an *a priori* power analysis revealed that 34 participants would yield 80.7% power at an α = 0.050.

Changes in body composition outcomes were evaluated using linear mixed-effects models with participant included as the random intercept to account for repeated observations across time. Fixed effects included time, sex, and initial weight status, and their interaction terms. Percent change (reductions) for each outcome variable, with the exception of BF%, was analyzed by sex and initial weight status groups using ANOVA. Bonferroni-Holm corrections were applied to all *post hoc* pairwise comparisons following significant omnibus effects to control family-wise error rates associated with multiple testing.

Initial weight status was categorized as upper (BMI ≥ 35 kg/m^2^; n = 14) and lower-BMI (BMI < 35 kg/m^2^; n = 20) based on the cutoff for class II obesity (see **Table 1**). The proportion of total weight loss attributable to FM, FFM, and SMM was calculated for each participant. In cases where FFM and SMM increased over the treatment period, weight loss was attributed entirely to FM (i.e., FM = 100%, FFM and SMM = 0%). Unless otherwise specified, all non-ratio body mass and fluid variables are reported in kilograms (kg), BF% is reported as a percentage of total body mass (%), and BMI is reported in kg/m2. Exceptions include outcomes expresses as a percent change from baseline and the proportion of total weight loss attributable to FM, FFM, or SMM. Data were analyzed using R. Statistical significance was set at p < 0.050, and data are presented as mean ± standard deviation.

**Table 1:** Body composition changes over the course of GLP-1RA therapy.

	Pre-therapy	Post-therapy			
N = 35	N (%)	N (%)			
Weight Status BMI ≥ 35 kg/m^2^	20 (57%)	1 (3%)			
Weight Status BMI < 35 kg/m^2^	15 (43%)	33 (97%)			
	Mean (95% CI)	Mean (95% CI)	Δ (95% CI)	%Δ (95% CI)	% of weight loss
Age (y)	53 (50, 56)				
Height (cm)	174 (171, 177)				
Weight (kg)	109.5 (101.3, 117.7)	78.0 (72.4, 83.6) †	-31.1 (-36.6, -25.7)	-27.8 (-31.2, -24.3)	85.7 (81.9,89.5)
BMI (kg/m^2^)	35.9 (33.6, 38.2)	25.5 (24.2, 26.8) †	-10.4 (-12.3, -8.5)	-27.8 (-31.3, -24.4)	13.8 (10.1, 17.4)
Body Fat (%)	42.5 (39.8, 45.3)	26.5 (24.5, 28.5) †	-16.2 (-18.1, -14.4)	-37.6 (-40.7, -34.4)	8.6 (6.2, 11.0)
Fat mass (kg)	47.0 (41.6, 52.4)	20.7 (18.6, 22.9) †	-26.4 (-30.9, -21.9)	-54.2 (-57.9, -50.5)	
Fat-free mass (kg)	62.5 (57.8, 67.2)	57.6 (52.9, 62.3) †	-4.5 (-5.9, -3.0)	-7.3 (-9.7, -4.8)	
SMM (kg)	34.9 (32.1, 37.7)	31.9 (29.0, 34.7) †	-2.8 (-3.7, -1.9)	-8.3 (-11.0, -5.6)	
SMM: Age	0.68 (0.61, 0.74)	0.62 (0.55, 0.68) †	-0.06 (-0.07, -0.04)	-8.3 (-11.0, -5.6)	
Dry lean mass (kg)	16.5 (15.3, 17.7)	15.4 (14.1, 16.7) †	-1.0 (-1.4, -0.6)	-6.2 (-8.6, -3.7)	
TBW (kg)	46.0 (42.5, 49.4)	42.2 (38.7, 45.7) †	-3.5 (-4.5, -2.4)	-7.7 (-10.1, -5.2)	
ECW (kg)	28.3 (26.2, 30.5)	25.6 (23.2, 27.9) †	-2.6 (-3.6, -1.5)	-9.5 (-13.5, -5.4)	
ICW (kg)	17.7 (16.3, 19.0)	16.2 (14.9, 17.5) †	-1.3 (-1.7, -0.9)	-7.4 (-9.8, -5.1)	
ECW: TBW	0.38 (0.38, 0.39)	0.39 (0.38, 0.39)	-0.00 (-0.00, 0.00)	0.14 (-0.64, 0.92)	
ECW: ICW	0.62 (0.62, 0.63)	0.63 (0.62, 0.64)	-0.00 (-0.01, 0.01)	0.27 (-0.96, 1.50)	

^†^significantly different from baseline (pre-therapy).

## Results

As shown in **Table 2**. Body weight, BMI, BF%, FM, FFM, SMM, SMM-to-age, DLM, TBW, ECW, and ICW all significantly declined over the course of GLP-1RA therapy (all p < 0.001). Indices of fluid distribution remained stable, with no significant changes observed in ECW: TBW or ECW: ICW (both p ≥ 0.674). Although absolute lean tissue measures decreased during treatment, weight loss was predominately attributable to reductions in FM (85.7 ± 10.9% of total weight loss from FM), with comparatively smaller contributions from FFM (13.8 ± 10.5% of total weight loss from FM) and SMM (8.6 ± 6.9 of total weight loss from FM).

**Table 2:** Body composition changes over the course of GLP-1RA therapy by sex.

	Male				Female			
	Pre-therapy	Post-therapy			Pre-therapy	Post-therapy		
N = 35	N (%)	N (%)			N (%)	N (%)		
Weight Status BMI ≥35 kg/m^2^	7 (47%)	1 (7%)			8 (40%)	0 (0%)		
Weight Status BMI <35 kg/m^2^	8 (53%)	13 (93%)			12 (60%)	20 (100%)		
	Mean (95% CI)	Mean (95% CI)	Δ (95% CI)	%Δ (95% CI)	Mean (95% CI)	Mean (95% CI)	Δ (95% CI)	%Δ (95% CI)
Age (y)	52 (48, 56)				54 (49, 59)			
Height (cm)	181 (177, 185)				169 (166, 172)			
Weight (kg)	121.6 (106.3, 137.0)	92.1 (84.7, 99.4)	-29.6 (-41.0, -18.2)	-22.5 (-28.2, -16.9) †	100.4 (93.1, 107.7)	68.2 (63.8, 72.6)	-32.2 (-38.2, -26.3)	-31.4 (-35.3, -27.5)
BMI (kg/m^2^)	37.2 (32.3, 42.0)	28.2 (26.0, 30.3)	-9.1 (-12.7, -5.5)	-22.6 (-28.2, -16.9) †	35.0 (32.8, 37.2)	23.6 (22.7, 24.6)	-11.3 (-13.4, -9.2)	-31.5 (-35.5, -27.6)
Body Fat (%)	38.1 (33.9, 42.4)	23.2 (20.4, 26.0)	-15.1 (-18.7, -11.5)	-38.5 (-44.6, -32.4)	45.8 (42.8, 48.9)	28.8 (26.5, 31.2)	-17.0 (-19.2, -14.9)	-36.9 (-40.6, -33.3)
Fat mass (kg)	47.9 (36.5, 59.3)	22.3 (17.6, 27.1)	-25.9 (-35.5, -16.2)	-50.8 (-58.0, -43.6)	46.3 (40.9, 51.8)	19.6 (17.6, 21.5)	-26.8 (-31.3, -22.2)	-56.6 (-60.6, -52.6)
FFM (kg)	73.7 (68.0, 79.5)	70.5 (65.8, 75.1)	-3.0 (-5.2, -0.8)	-3.6 (-6.5, -0.7) †	54.1 (49.9, 58.2)	48.6 (44.7, 52.5)	-5.5 (-7.4, -3.5)	-9.8 (-13.2, -6.5)
SMM (kg)	41.6 (38.3, 45.0)	39.6 (36.9, 42.4)	-1.9 (-3.3, -0.5)	-4.0 (-7.3, -0.7) †	29.9 (27.4, 32.4)	26.4 (24.1, 28.8)	-3.5 (-4.7, -2.3)	-11.3 (-15.0, -7.6)
SMM: Age	0.81 (0.74, 0.9)	0.77 (0.71, 0.84)	-0.04 (-0.06, -0.01)	-4.0 (-7.3, -0.7) †	0.58 (0.51, 0.64)	0.51 (0.45, 0.56)	-0.1 (-0.1, -0.01)	-11.3 (-15.0, -7.6)
DLM (kg)	19.5 (18.0, 21.0)	18.8 (17.6, 20.1)	-0.58 (-1.2, 0.02)	-2.5 (-5.5, 0.5) †	14.3 (13.2, 15.4)	13.0 (12.0, 14.0)	-1.3 (-1.8, -0.8)	-8.7 (-12.1, -5.4)
TBW (kg)	54.3 (50.0, 58.5)	51.6 (48.2, 55.0)	-2.5 (-4.1, -0.8)	-4.1 (-7.0, -1.1) †	39.8 (36.7, 42.8)	35.6 (32.7, 38.5)	-4.2 (-5.6, -2.7)	-10.2 (-13.6, -6.9)
ECW (kg)	33.5 (30.9, 36.0)	31.9 (29.8, 34.0)	-1.5 (-2.5, -0.4)	-3.9 (-7.0, -0.7) †	24.5 (22.5, 26.4)	21.1 (18.9, 23.3)	-3.4 (-4.9, -1.8)	-13.4 (-19.6, -7.2)
ICW (kg)	20.8 (19.1, 22.5)	19.7 (18.3, 21.1)	-1.0 (-1.7, -0.4)	-4.4 (-7.2, -1.6) †	15.3 (14.2, 16.5)	13.8 (12.7, 14.9)	-1.5 (-2.0, -1.0)	-9.5 (-12.8, -6.3)
ECW: TBW	0.38 (0.38, 0.39)	0.38 (0.37, 0.39) †	-0.00 (-0.01, 0.00)	-0.9 (-2.5, 0.8) †	0.39 (0.38, 0.39)	0.39 (0.39, 0.39)	0.00 (0.00, 0.01)	0.85 (0.21, 1.5)
ECW: ICW	0.62 (0.61, 0.63)	0.61 (0.59, 0.63) †	-0.01 (-0.03, 0.01)	-1.3 (-3.9, 1.2) †	0.63 (0.62, 0.64)	0.64 (0.63, 0.65)	0.01 (0.00, 0.02)	1.4 (0.35, 2.5)

^†^significantly different from females.

Significant sex by time interactions were observed for ECW: TBW and ECW: ICW (both p = 0.022). *Post hoc* tests revealed that males exhibited significantly lower ECW: TBW (p = 0.015) and ECW: ICW (p = 0.017) following treatment when compared to females. Percent reductions (%Δ) in body weight, BMI, FFM, SMM, SMM-to-age, DLM, TBW, ECW, and ICW were significantly greater in females (all p ≤ 0.025). Conversely, percent reductions (%Δ) in ECW: TBW (p = 0.025) and ECW: ICW (p = 0.023) were significantly greater in males. No significant sex differences were observed for percent change (%Δ) in FM (p = 0.114) or in the proportion of total weight loss attributable to FM, FFM, or SMM (all p ≥ 0.140).

Significant initial weight status by time interactions were observed for BF% (p = 0.004), body weight, BMI, FM, FFM, SMM, SMM-to-age, DLM, TBW, ECW, and ICW (all p < 0.001). Prior to treatment, weight, BMI, BF%, and FM were significantly higher in the upper-BMI group compared with the lower BMI group (p ≤ 0.003), insert see **Table 3**. Although both groups experienced significant reductions in these variables following treatment, no between group differences were observed at the post-treatment timepoint (p ≥ 0.115).

**Table 3:** Body composition changes over the course of GLP-1RA therapy by initial weight status.

	Lower BMI				Upper BMI			
	Pre-therapy	Post-therapy			Pre-therapy	Post-therapy		
	Mean (95% CI)	Mean (95% CI)	Δ (95% CI)	%Δ (95% CI)	Mean (95% CI)	Mean (95% CI)	Δ (95% CI)	%Δ (95% CI)
Age (y)	53 (48, 57)				53 (49, 58)			
Height (cm)	173 (169, 178)				176 (171, 180)			
Weight (kg)	95.5 (90.3, 100.7) †	74.5 (67.9, 81.2) ‡	-20.9 (-23.8, -18.0)	-22.4 (-25.9, -18.9) †	128.2 (115.3, 141.2)	83.0 (72.7, 93.2) ‡	-45.7 (-53.5, -37.9)	-35.4 (-39.9, -31.0)
BMI (kg/m2)	31.8 (30.8, 32.7) †	24.6 (23.3, 25.9) ‡	-7.2 (-8.4, -6.0)	-22.4 (-25.9, -18.9) †	41.4 (37.7, 45.2)	26.8 (24.2, 29.3) ‡	-15.0 (-17.8, -12.3)	-35.6 (-40.0, -31.1)
Body Fat (%)	39.4 (35.9, 42.8) †	25.3 (22.9, 27.8) ‡	-14.0 (-16.0, -12.0)	-35.4 (-38.9, -31.9)	46.8 (43.0, 50.5)	28.2 (24.8, 31.5) ‡	-19.4 (-22.5, -16.3)	-40.7 (-46.6, -34.7)
Fat mass (kg)	37.1 (34.6, 39.5) †	18.5 (17.0, 20.0) ‡	-18.6 (-20.8, -16.4)	-49.8 (-53.6, -45.9) †	60.3 (51.7, 68.9)	23.9 (19.4, 28.5) ‡	-37.6 (-44.8, -30.3)	-60.5 (-66.6, -54.5)
FFM (kg)	58.4 (52.4, 64.4) †	56.1 (49.8, 62.3) ‡	-2.3 (-3.9, -0.8)	-4.2 (-7.0, -1.5) †	67.9 (60.6, 75.2)	59.8 (51.6, 67.9) ‡	-7.5 (-9.4, -5.5)	-11.6 (-15.3, -7.9)
SMM (kg)	32.6 (28.9, 36.2) †	31.1 (27.3, 34.8) ‡	-1.5 (-2.5, -0.6)	-4.9 (-8.0, -1.9) †	38.1 (33.8, 42.4)	33.0 (28.1, 37.9) ‡	-4.7 (-5.9, -3.5)	-13.1 (-17.3, -8.9)
SMM: Age	0.64 (0.55, 0.73) †	0.61 (0.52, 0.69) ‡	-0.03 (-0.05, -0.01)	-4.9 (-8.0, -1.9) †	0.73 (0.63, 0.82)	0.63 (0.52, 0.73) ‡	-0.1 (-0.1, -0.07)	-13.1 (-17.3, -8.9)
DLM (kg)	15.5 (13.9, 17.1) †	15.0 (13.4, 16.7)	-0.5 (-0.9, -0.07)	-3.3 (-6.2, -0.5) †	17.8 (15.9, 19.7)	15.9 (13.8, 18.1) ‡	-1.7 (-2.3, -1.1)	-10.2 (-14.0, -6.4)
TBW (kg)	42.9 (38.5, 47.3) †	41.1 (36.5, 45.7) ‡	-1.8 (-3.0, -0.7)	-4.6 (-7.3, -1.8) †	50.1 (44.7, 55.5)	43.8 (37.8, 49.8) ‡	-5.8 (-7.2, -4.4)	-12.2 (-15.8, -8.6)
ECW (kg)	26.5 (23.7, 29.3) †	25.3 (22.4, 28.2)	-1.2 (-1.9, -0.4)	-4.7 (-7.5, -1.8) †	30.7 (27.4, 34.0)	25.9 (21.3, 30.4) ‡	-4.6 (-6.5, -2.6)	-16.3 (-24.5, -8.1)
ICW (kg)	16.4 (14.8, 18.0) †	15.7 (14.0, 17.4) ‡	-0.7 (-1.1, -0.3)	-4.4 (-7.0, -1.8) †	19.3 (17.3, 21.4)	17.0 (14.7, 19.2) ‡	-2.2 (-2.7, -1.7)	-11.8 (-15.0, -8.6)
ECW: TBW	0.38 (0.38, 0.39)	0.38 (0.38, 0.39)	-0.00 (-0.00, -0.00)	0.28 (-0.33, 0.89)	0.39 (0.38, 0.39)	0.39 (0.38, 0.39) ‡	-0.00 (-0.01, 0.01)	-0.06 (-1.9, -1.8)
ECW: ICW	0.62 (0.61, 0.63)	0.62 (0.61, 0.63)	-0.00 (-0.00, -0.01)	0.48 (-0.51, 1.5)	0.63 (0.62, 0.64)	0.63 (0.61, 0.65) ‡	-0.00 (-0.02, 0.02)	-0.02 (-2.9, -2.9)

^†^significantly different from upper BMI group at the corresponding timepoint.

^‡^significantly different from pre-treatment within group.

FFM, SMM, TBW, and ICW decreased significantly in both BMI groups (all p ≤ 0.021) but did not differ between groups at either baseline or post-treatment (p ≥ 0.074). In contrast, DLM and ECW decreased significantly in the upper-BMI group (all p < 0.001) but not in the lower BMI group (all p ≥ 0.126); however, neither variable differed between groups at baseline or post treatment (all p ≥ 0.185). No significant initial weight status by time interactions were observed for ECW: TBW or ECW: TBW (both p ≥ 0.650).

As Shown in **Table 3**. Percent reductions (%Δ) in body weight, BMI, FM, FFM, SMM, SMM-to-age, DLM, TBW, ECW, and ICW were significantly greater in the upper-BMI group compared with the lower BMI group (all p ≤ 0.004). Conversely, percent reductions in ECW: TBW and ECW: ICW (both p ≥ 0.663), as well as the proportion of total weight loss attributable to FM, FFM, and SMM (all p ≥ 0.071), did not differ by initial weight status groups.

There were no other significant interactions between time, sex, or initial weight status for any other variable, including percent change (all p ≥ 0.063).

## Discussion

This retrospective study examined body weight and bioelectrical impedance analysis derived body composition changes in adults with overweight and obesity treated with a dual glucose dependent insulinotropic polypeptide and glucagon like peptide 1 receptor agonist tirzepatide in a physician managed real world clinical setting. The primary findings were that weight, BMI, body fat percentage, fat mass, fat free mass, skeletal muscle mass, total body water, extracellular water, and intracellular water all declined significantly, while indices of fluid distribution remained stable. Weight loss was predominantly attributable to fat mass reduction, with smaller contributions from fat free mass and skeletal muscle mass. In addition, sex and baseline weight status influenced several outcomes, with females demonstrating greater percentage reductions across multiple compartments and individuals with higher baseline BMI showing larger percentage reductions overall, without differences in the proportional contribution of fat mass, fat free mass, or skeletal muscle mass to total weight loss. These findings indicate that treatment with a dual GIP and GLP 1 receptor agonist in routine clinical care produces substantial weight loss driven primarily by adipose tissue reduction while generally preserving proportional fluid distribution.

The predominance of fat mass loss is consistent with the physiological actions of incretin based therapies. Glucagon like peptide 1 receptor activation increases satiety, reduces energy intake, delays gastric emptying, and improves glucose dependent insulin secretion (8). Tirzepatide, as a dual GIP and GLP 1 receptor agonist, further augments metabolic regulation by enhancing insulin sensitivity and appetite suppression through complementary pathways (10, 11). These mechanisms promote sustained negative energy balance and preferential mobilization of adipose tissue. Given the strong association between excess adiposity and cardiometabolic risk, reductions in fat mass may yield clinically meaningful improvements in metabolic health that are not fully captured by total body weight alone (18). The observed magnitude of fat mass and body fat percentage reduction supports the clinical value of these therapies in shifting patients toward a more favorable body composition profile in real world practice settings (6, 7).

Despite the predominance of fat mass loss, reductions in fat free mass and skeletal muscle mass were also observed and remain clinically relevant. These changes likely reflect multiple physiological factors associated with weight loss, including reduced mechanical loading, decreased anabolic signaling, and potential inadequacies in protein intake or resistance exercise. In addition, improvements in glycemic control and changes in substrate utilization can alter muscle glycogen stores and associated water content, contributing to reductions in fat free mass and total body water measured using impedance methods. Loss of lean tissue has important implications, including potential reductions in resting metabolic rate, functional capacity, and long term weight maintenance, and is associated with adverse outcomes in sarcopenic obesity (13, 14). These findings reinforce the importance of integrating nutritional and exercise strategies to preserve skeletal muscle during pharmacologically induced weight loss.

Changes in body water compartments provide additional insight into tissue-level adaptations during weight loss. Significant reductions were observed in total body water (TBW), extracellular water (ECW), and intracellular water (ICW); however, the stability of ECW: TBW and ECW: ICW ratios indicates that fluid distribution remained proportionate over time despite overall declines in fluid volume. This pattern suggests that reductions in water compartments were primarily driven by overall mass loss and associated decreases in lean tissue and glycogen-bound water, rather than disproportionate fluid shifts or alterations in hydration status. Observed sex-related differences in fluid indices, with males demonstrating greater changes in fluid ratios and females showing larger percentage reductions in absolute compartments, may reflect underlying physiological differences in body water distribution and hormonal influences.

Additionally, sex and baseline BMI moderated several percent change outcomes, reinforcing the importance of individualized monitoring and counseling when interpreting body composition changes. Together, these findings highlight the need to consider patient-specific characteristics when evaluating bioelectrical impedance analysis–derived measures during treatment. Overall, the results support the clinical value of integrating practical body composition assessment into GLP-1–based obesity management to maximize adipose tissue reduction while proactively preserving lean mass and function, thereby improving metabolic health outcomes in real-world practice (8, 18).The present findings are consistent with evidence from randomized trials demonstrating significant weight loss with incretin-based therapies. Semaglutide has been associated with average weight reductions of approximately 15–16%, while tirzepatide has produced reductions of up to 22.5% in the SURMOUNT-1 trial (5, 9). Although differences in study design, duration, and patient populations limit direct comparisons, the magnitude of weight loss observed in this cohort supports the effectiveness of dual GIP/GLP-1 receptor agonist therapy in real-world settings. Importantly, this study extends prior work by providing a detailed evaluation of multi-compartment body composition changes rather than relying solely on weight-based outcomes, which has been identified as a limitation in obesity treatment research (6, 7).

These findings are further supported by a recent systematic review and meta-analysis by Sawicka-Gutaj et al. (19), which demonstrated that after 12 months of GLP-1 receptor agonist (GLP-1RA) therapy, significant improvements were observed in body composition, including a 4% reduction in fat mass (RoM 0.96; 95% CI: 0.92–0.99; p < 0.001) and a parallel 4% reduction in lean body mass (RoM 0.96; 95% CI: 0.95–0.97; p < 0.001), although substantial intergroup heterogeneity was noted. These results highlight that while GLP-1RAs effectively reduce adiposity, concomitant losses in lean mass are also common, reinforcing the importance of comprehensive body composition assessment.

The proportional contribution of fat mass to total weight loss observed in this study aligns with emerging clinical trial data. In the SURMOUNT-1 body composition substudy, approximately 75% of weight loss was attributed to fat mass and 25% to lean mass over 72 weeks (15). The present findings demonstrate a similar pattern, with fat mass accounting for the majority of weight reduction, while also emphasizing that lean tissue loss remains clinically meaningful. Studies using bioelectrical impedance analysis in clinical populations similarly report substantial reductions in fat mass alongside relatively preserved cellular integrity markers and improvements in metabolic health (16, 17).

Collectively, these data indicate that while adipose tissue reduction is the primary driver of weight loss with incretin-based therapies, concurrent reductions in lean mass are consistently observed across studies. This underscores the importance of incorporating detailed body composition and metabolic phenotyping into obesity treatment research to better understand the physiological adaptations to therapy and to inform strategies aimed at preserving lean tissue while maximizing fat loss. GIP and GLP 1 receptor agonist.

These results have several practical implications for clinical obesity management. Demonstrating that weight loss is largely attributable to fat mass may support patient understanding of treatment benefits and improve adherence (6, 18). At the same time, measurable reductions in fat free mass and skeletal muscle mass highlight the importance of routine body composition assessment in clinical practice. Bioelectrical impedance analysis offers a feasible approach for tracking these changes and identifying individuals at risk for excessive lean tissue loss, allowing for timely intervention through nutritional optimization and resistance training (13, 14). The stability of fluid distribution indices provides additional reassurance that observed changes reflect true compositional shifts rather than major alterations in hydration balance. Finally, sex and baseline BMI related differences suggest that individualized interpretation and management strategies may enhance treatment outcomes.

This study should be interpreted in the context of several important limitations. First, the retrospective observational design precludes causal inference and limits the ability to control for key confounding variables that may influence body composition changes. Specifically, we were unable to account for dietary factors most notably protein intake which plays a critical role in preserving fat-free mass and skeletal muscle during weight loss. Similarly, participation in resistance exercise or structured physical activity was not systematically quantified, despite its well-established importance in mitigating lean tissue loss. Variability in medication dosing, titration schedules, and adherence behaviors further introduces heterogeneity that may affect individual responses to therapy with dual GIP/GLP-1 receptor agonists (6, 7).

Second, the absence of functional outcome measures limits the clinical interpretation of observed changes in body composition. While reductions in fat-free mass (kg) and skeletal muscle mass (kg) were quantified, the study did not include assessments of muscle strength (e.g., handgrip strength), physical performance (e.g., gait speed, chair rise time), or patient-reported functional status. As a result, it is not possible to determine whether reductions in lean tissue translated into meaningful impairments or were offset by improvements in physical capacity or quality of life. This distinction is particularly relevant in the context of sarcopenic obesity, where preservation of function may be more clinically important than absolute tissue mass (14).

Third, the relatively small sample size and single-center design limit statistical power and generalizability. The cohort may not fully represent the broader population of individuals receiving dual GIP/GLP-1 receptor agonist therapy across diverse demographic, clinical, and socioeconomic contexts. In addition, subgroup analyses (e.g., by sex and baseline BMI) should be considered exploratory, as the study was not specifically powered for these comparisons. Accordingly, the findings should be interpreted as real-world, hypothesis-generating evidence rather than definitive clinical conclusions.

A major methodological limitation relates to the use of bioelectrical impedance analysis (BIA) for body composition assessment. Although BIA is practical, noninvasive, and widely accessible in outpatient settings, it is inherently indirect and relies on prediction equations to estimate fat mass (kg), fat-free mass (kg), and total body water (kg) compartments. Importantly, BIA measurements are highly sensitive to physiological and behavioral factors that influence fluid balance, including hydration status, recent food or fluid intake, glycogen depletion, and acute or chronic shifts in extracellular and intracellular water. These considerations are particularly relevant in patients undergoing treatment with dual GIP/GLP-1 receptor agonists, which may alter appetite, nutrient intake, and hydration behaviors.

While the observed stability in fluid distribution indices (e.g., ECW: TBW, ECW: ICW) provides some reassurance regarding measurement consistency at the group level, these indices cannot fully eliminate concern regarding compartment misclassification at the individual level. Changes attributed to fat-free mass or skeletal muscle mass may, in part, reflect alterations in body water distribution rather than true changes in contractile tissue. This limitation is especially pertinent given that BIA assesses fat-free mass at the molecular level, whereas gold-standard imaging techniques such as dual-energy X-ray absorptiometry (DXA), computed tomography (CT), and magnetic resonance imaging (MRI) provide more direct estimates of muscle mass and regional tissue composition (Tinsley et al., (20)). As such, BIA-derived estimates of lean mass should be interpreted with caution, and the potential for overinterpretation of compartment-specific changes must be acknowledged.

Finally, the study did not include direct imaging assessments of visceral adiposity or bone mineral density, which limits the ability to fully characterize the cardiometabolic and musculoskeletal implications of weight loss (14, 18). In addition, emerging perspectives emphasize the importance of evaluating safety considerations related to changes in fat-free mass (FFM). FFM comprises muscle, bone, and other non-fat tissues, and reductions in these components particularly skeletal muscle and bone have been associated with increased risk of frailty, osteoporosis, falls, and fractures (Bennett & Lim, (21)). Notably, declines in FFM have also been independently linked to higher risk of all-cause, cardiovascular, and cancer-related mortality, underscoring the clinical importance of monitoring these changes during weight loss interventions (Bennett & Lim, (21)). Without concurrent assessment of bone density, muscle strength, and physical performance, it remains unclear whether observed reductions in lean mass reflect meaningful functional decline or largely benign changes in tissue composition.

Taken together, these limitations underscore that the present findings represent exploratory, real-world evidence describing body composition changes during dual GIP/GLP-1 receptor agonist therapy. Future prospective studies incorporating larger, more diverse cohorts; standardized assessments of diet and physical activity, including protein intake and resistance exercise; direct imaging modalities; and objective functional outcomes (e.g., strength, gait speed, chair rise performance) are needed to more definitively characterize the clinical significance of these changes. Expanding real-world investigations to include these measures will help clarify the balance between fat loss and preservation of musculoskeletal health, particularly in populations at higher risk for adverse functional outcomes. Stratified analyses by medication type (semaglutide vs. tirzepatide), baseline metabolic status (e.g., presence of type 2 diabetes), and sex may further elucidate differential response patterns suggested by current findings (5, 11).

Finally, pairing BIA with imaging substudies and longer follow-up would help confirm whether early adiposity reductions are sustained and whether lean mass trajectories stabilize with targeted lifestyle support, aligning clinical practice with best-practice recommendations for metabolic health optimization (16, 17). In conclusion, treatment with dual glucose-dependent insulinotropic polypeptide/glucagon-like peptide-1 (GIP/GLP-1) receptor agonists in routine physician-managed obesity care resulted in significant weight loss and substantial improvements in body composition, with most of the weight reduction driven by decreases in fat mass and smaller yet meaningful reductions in lean tissue compartments.

## Data Availability

The original contributions presented in the study are included in the article/supplementary material. Further inquiries can be directed to the corresponding author.
